# Hormone‐mediated foraging strategies in an uncertain environment: Insights into the at‐sea behavior of a marine predator

**DOI:** 10.1002/ece3.7590

**Published:** 2021-05-03

**Authors:** Eugene J. DeRango, Jonas F. L. Schwarz, Paolo Piedrahita, Diego Páez‐Rosas, Daniel E. Crocker, Oliver Krüger

**Affiliations:** ^1^ Department of Animal Behaviour Bielefeld University Bielefeld Germany; ^2^ Facultad de Ciencias de la Vida Escuela Superior Politécnica del Litoral Guayaquil Ecuador; ^3^ Universidad San Francisco de Quito Galápagos Science Center Isla San Cristobal Ecuador; ^4^ Dirección Parque Nacional Galápagos Oficina Técnica San Cristóbal Isla San Cristóbal Ecuador; ^5^ Department of Biology Sonoma State University Rohnert Park CA USA

**Keywords:** cortisol, diving behavior, Galapagos sea lion, repeatability, testosterone, thyroid

## Abstract

1. Hormones are extensively known to be physiological mediators of energy mobilization and allow animals to adjust behavioral performance in response to their environment, especially within a foraging context.
2. Few studies, however, have narrowed focus toward the consistency of hormonal patterns and their impact on individual foraging behavior. Describing these relationships can further our understanding of how individuals cope with heterogeneous environments and exploit different ecological niches.
3. To address this, we measured between‐ and within‐individual variation of basal cortisol (CORT), thyroid hormone T3, and testosterone (TEST) levels in wild adult female Galápagos sea lions (*Zalophus wollebaeki*) and analyzed how these hormones may be associated with foraging strategies. In this marine predator, females exhibit one of three spatially and temporally distinct foraging patterns (i.e., “benthic,” “pelagic,” and “night” divers) within diverse habitat types.
4. Night divers differentiated from other strategies by having lower T3 levels. Considering metabolic costs, night divers may represent an energetically conservative strategy with shorter dive durations, depths, and descent rates to exploit prey which migrate up the water column based on vertical diel patterns.
5. Intriguingly, CORT and TEST levels were highest in benthic divers, a strategy characterized by congregating around limited, shallow seafloors to specialize on confined yet reliable prey. This pattern may reflect hormone‐mediated behavioral responses to specific risks in these habitats, such as high competition with conspecifics, prey predictability, or greater risks of predation.
6. Overall, our study highlights the collective effects of hormonal and ecological variation on marine foraging. In doing so, we provide insights into how mechanistic constraints and environmental pressures may facilitate individual specialization in adaptive behavior in wild populations.

## Graphical abstract

Hormones are known to be physiological mediators of energy mobilization and behavioral adjustment during foraging episodes; however, few studies have narrowed focus and described this relationship on an intraspecific level. We show strong between‐individual variation of cortisol, testosterone, and thyroid hormones in adult female Galapagos sea lions, which align with highly diverse foraging strategies. In doing so, we provide insights into how mechanistic constraints and risk assessment may facilitate individual specialization in foraging behavior to exploit different ecological niches.

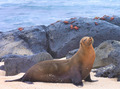

## 1 BACKGROUND

Hormonal axes have long been understood to mediate essential physiological systems in animals, with glucocorticoids (specifically cortisol, or CORT, in mammals) contributing to an adaptive ability to maintain homeostasis and regulate a multitude of biological functions (Crespi et al., 2013; Romero & Wingfield, 2016; Wingfield & Kitaysky, 2002). According to the “energy mobilization hypothesis,” CORT, in tandem with other endocrine systems such as thyroid hormones (Castañeda Cortés et al., 2014; McNabb & King, 1993), is a driving force behind cellular metabolic shifts which are crucial in coping with the demands of environmental stressors (Romero, 2002). Concisely, these hormones are released to maintain homeostasis when energy requirements exceed available energy (e.g., during nutritional duress, harsh weather, predator exposure; Sapolsky et al., 2000; Romero, 2002). In that regard, traditional frameworks suggest these hormones are responsible for the phenotypic organization of behavior and support responses toward the current environmental landscape (Hau & Goymann, 2015; Landys et al., 2006; Romero, 2002). Less understood, however, is how variation in hormonal axes and stress response mechanisms may prove adaptive under this selection and drive behavioral trait combinations often found across or within species in nature. Therefore, a key first step is describing hormonal‐behavioral associations within the context of ecological pressures to determine the mechanisms and trade‐offs behind this variation (Taborsky et al., 2020).

A focus on foraging behavior may help elucidate these questions, as it is an integral and demanding aspect of life history that is tightly regulated by trade‐offs between the acquisition and investment of energy (Arvidsson & Matthysen, 2016; Schoener, 1971). Experimental and comparative studies have shown that elevation of hormones, such as glucocorticoids, has broad effects on locomotion, such as by increasing the number of food visitation bouts and foraging efficiency for songbirds (Lohmus et al., 2006; Pravosudov, 2003) or altering diving and prey‐chasing behavior in several species of seabirds, such as penguins and albatross (Angelier et al., 2007, 2008; Cottin et al., 2014; Crossin et al., 2012; Kroeger et al., 2019). In a separate fashion, androgens, such as testosterone (TEST), are classically known to increase competition or territory defense among conspecifics (Mehta & Josephs, 2010; Wingfield et al., 1990), but have also been linked to the aggressiveness of search behavior toward prey (Desprat et al., 2017) and vigilance toward conspecifics while foraging in group contexts (Kellam et al., 2006).

While these hormonal effects are often considered species‐level phenomena, there has been a paradigm shift within behavioral endocrinology toward recognizing hormonal variation on the individual level. Efforts now strongly emphasize the consistency (i.e., repeatability) of hormones between and within individuals (Cockrem, 2013; Hau et al., 2016; Taff et al., 2018). Closely intertwined is the field of “animal personality,” which similarly describes consistent individual differences in behavior within or across ecologically relevant timescales and contexts (Réale et al., 2010; Sih et al., 2015; Wolf & Weissing, 2010). Foraging behavior receives particular attention, as it often encompasses strong interindividual variation in risk assessment, activity level, and sociability, with each having strong impacts on the fitness of an individual (reviewed in Toscano et al., 2016). A growing body of evidence suggests that variation in physiological components, such as hormones or general metabolic constraints, underlies general individual differences (Biro & Stamps, 2010; Careau et al., 2008; Sih et al., 2015) and likely regulates the phenotypic expression and consistency of these behaviors (Baugh et al., 2017; Dammhahn et al., 2018). In this regard, integrating the study of the physiological ecology and behavioral repertoires of wild individual animals is an exciting, although relatively unexplored, avenue to contextualize intraspecific diversity in foraging behavior.

Galápagos sea lions (GSL, *Zalophus wollebaeki*) are an intriguing model species to consider these effects. Like all eared seals (family Otariidae), GSL are central place foragers, wherein terrestrial breeding and marine feeding occur in confined geographic ranges centered around seasonal productivity hotspots (Costa et al., 2004; Trillmich et al., 2014). This naturally creates strong site fidelity and a huge potential for spatial and temporal overlap of individuals (Páez‐Rosas & Aurioles‐Gamboa, 2014; Trillmich et al., 2014). These conditions favor high intraspecific diversity in diving behavior to alleviate competition within the marine environment (Páez‐Rosas & Aurioles‐Gamboa, 2014; Páez‐Rosas et al., 2017; Schwarz et al., 2021; Villegas‐Amtmann et al., 2008). In one centrally located population in the Galápagos archipelago, three distinct foraging strategies have been revealed with clear spatial and temporal separation and indications for stability of these strategies based on individual foraging episodes (Schwarz et al., 2021; Villegas‐Amtmann et al., 2008). Benthic (or bottom) diving females congregate around shallow seafloors at the coast or on top of underwater mountains, mostly showing a high fidelity toward solitary and relatively sessile benthic fish, which are presumed to be reliable but less nutrient‐dense food resources (Schwarz et al., 2021). Pelagic (or deep) divers, on the other hand, disperse toward highly mobile, high‐quality prey patches that require extensive searching and deep diving in open water between islands. A third strategy, the night divers, similarly search for schooling prey patches but generally do so in shallow depths during the night, when deep‐sea fish follow diel vertical migration and travel upwards within the water column (Hays, 2003). Studies have previously suggested inherent metabolic constraints underlying behavioral repertoires of individual GSL. For example, increased blood oxygen and myoglobin levels were found to be key contributors to a physiological capacity to extend dive durations in adult females (Villegas‐Amtmann & Costa, 2010), while time spent at sea was associated with increased field metabolic rates (FMRs; Villegas‐Amtmann et al., 2017).

Here, we further investigated physiological mechanisms enabling foraging strategies found in GSL by shedding light on the involvement of endocrine hormones as facilitators of individual differentiation in marine foraging. Because each strategy has been established to be unique in behavior and ecological pressures, we predicted strong between‐individual variation in hormone levels within the study population and intrinsic differences in hormone levels across each foraging group. To test this, we first quantified individual repeatability estimates of near‐baseline CORT, TEST, and thyroid T3 (triiodothyronine) before and after a two‐week window during which we remotely monitored foraging behavior. Using general linear models, we then examined the effects of hormone levels on foraging strategy type, while controlling for intrinsic and environmental variables that may impact hormone expression, including female age and mass, pup mass, and annual effects. By combining hormonal and behavioral datasets, this study thereby attempts to uncover proximate mechanisms and selective pressures to provide a deeper understanding of individual specialization in foraging strategies.

## 2 MATERIALS AND METHODS

### 2.1 Field site and procedures

**FIGURE 1:**
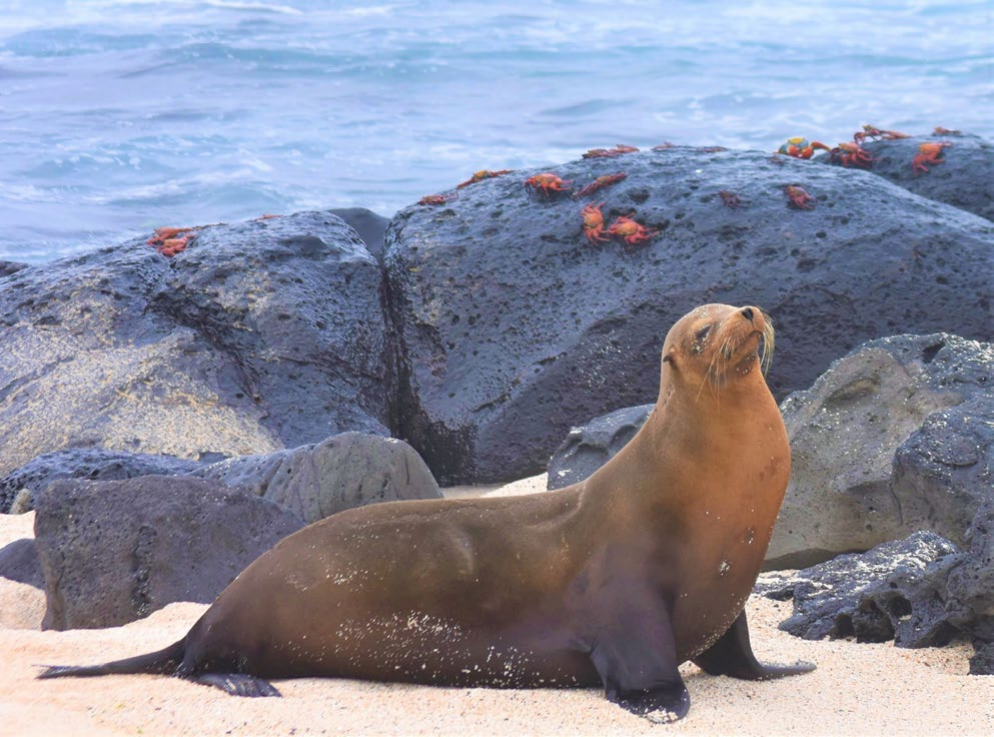
Adult female Galapagos sea lion (*Zalophus wollebaeki*). Photo credit: E. DeRango

This study used data from 34 adult female Galapagos sea lions with clearly distinct foraging strategy clusters (Figure 1), as defined by Schwarz et al. (2021). Here, distinct refers to individuals where all or all but one foraging trip fell into a known foraging strategy (described in greater detail below). Research was carried out on Caamaño, a small islet near Puerto Ayora, Santa Cruz Island (0°45′S, 90°16′W). Adult female sea lions were captured during October‐November 2018 (*N* = 15) and 2019 (*N* = 19), coinciding with peak reproductive activity (Trillmich et al., 2014). Using census data within a long‐term study, we determined the exact age of known individuals (*N* = 31), revealing a mean age of 13.5 years ± 3.7 *SD* (range of 6–19 years). Because of the extremely long dependency period of GSL (Trillmich et al., 2014), all females were observed to be actively nursing either a newly born or yearling pup. This large variation in pup size, and thus maternal effort, is a possible driver of foraging variation in this species (Villegas‐Amtmann et al., 2012) and was later considered in models which predict foraging strategy clusters.

Sea lions were captured in early morning using custom hoop nets (Fuhrman Diversified). Animals were resting and unaware of the capturer; therefore, chase was minimal. After restraint, venipuncture was attempted from the caudal gluteal vein using a 1.5 inch, 20 G vacutainer needle. A blood sample was quickly collected after initial disturbance (mean 2.2 min ± 0.7 *SD*) with the intention to measure near‐baseline hormonal values (Romero & Reed, 2005; Sapolsky et al., 2000). We then measured mass using a hanging sling and digital scale and affixed a biologging device (MK10 time‐depth recorder; Wildlife Computers) on the dorsum. We used a similar method to measure mass in dependent pups. Mass data for each animal were collated based on the most recent capture from the time of its mother's tagging date to best reflect pup mass at the time of foraging. To retrieve biologgers from females, animals were captured when first resighted upon return to the colony, or 16 days ± 0.7 *SD* after the initial capture (range 13–22 days). During this recapture, we collected a second blood sample within a similar time frame (mean 2.0 min ± 0.9 *SD*) to calculate repeatability of all hormones. Due to logistical reasons, this second sample was only collected in a random subset of females (*N* = 20).

### 2.2 Hormone analysis

Blood samples were centrifuged in the field at ~1,500 *g* for 15 min to separate serum. Due to lack of a freezer in remote conditions, serum was diluted 1:1 via pipette with pure ethanol until stored at −80°C. Ethanol preserves lipophilic hormones within serum with minimal degradation over time (Goymann et al., 2007) and has been used successfully in field conditions with pinniped serum (DeRango, Greig, et al., 2019). In the laboratory, we centrifuged samples again to separate the ethanol supernatant containing dissolved hormones for analysis.

All hormone measurements were quantified in duplicate using commercially available assays during a single run. Total CORT and total thyroid T3 (TT3) were measured using a hormone‐specific I^125^ RIA coated tube kit, and TEST was measured using an enzyme immunoassay platform (MP Biomedicals). Validations of these kits for GSL, including recovery and parallelism techniques, were previously described in DeRango et al. (2020).

### 2.3 At‐sea foraging behavior

The identification of foraging strategies was done by Schwarz et al. (2021) based on collected dive data. To summarize, putative foraging episodes of dives were identified by dividing dives into segments with an automated broken stick algorithm and focusing on a vertical restricted search area (Heerah et al., 2014). The mean of putative foraging durations, depths, depth ranges, and percentage of night dives for each foraging trip was calculated, as well as mean dive time and descent rates across all diving episodes (Table 1).

**TABLE 1:** Means and standard deviations of diving parameters for Galapagos sea lions arranged by foraging strategy cluster. Significant differences between foraging clusters are denoted by dissimilar letters

Parameter	Cluster 1 (Benthic) *N* = 12	Cluster 2 (Pelagic) *N* = 12	Cluster 3 (Night) *N* = 10
Foraging depth (m)	67.7 ± 24.2^a^	133.2 ± 18.4^b^	60.2 ± 31.2^a^
Foraging duration (s)	113.2 ± 32.8^a^	124.2 ± 33.4^a^	64.3 ± 19.2^b^
Foraging depth range (m)	3.38 ± 1.09^a^	4.89 ± 1.81^b^	4.55 ± 1.37^b^
Night dives (%)	15.9 ± 10.8^a^	17.8 ± 8.66^a^	68.9 ± 21.7^b^
Mean dive time (s)	187.7 ± 76.0^a^	275.4 ± 54.9^b^	154.5 ± 33.6^a^
Descent rate (m/s)	1.38 ± 0.30^a^	1.59 ± 0.15^b^	1.32 ± 0.14^a^

**FIGURE 2:**
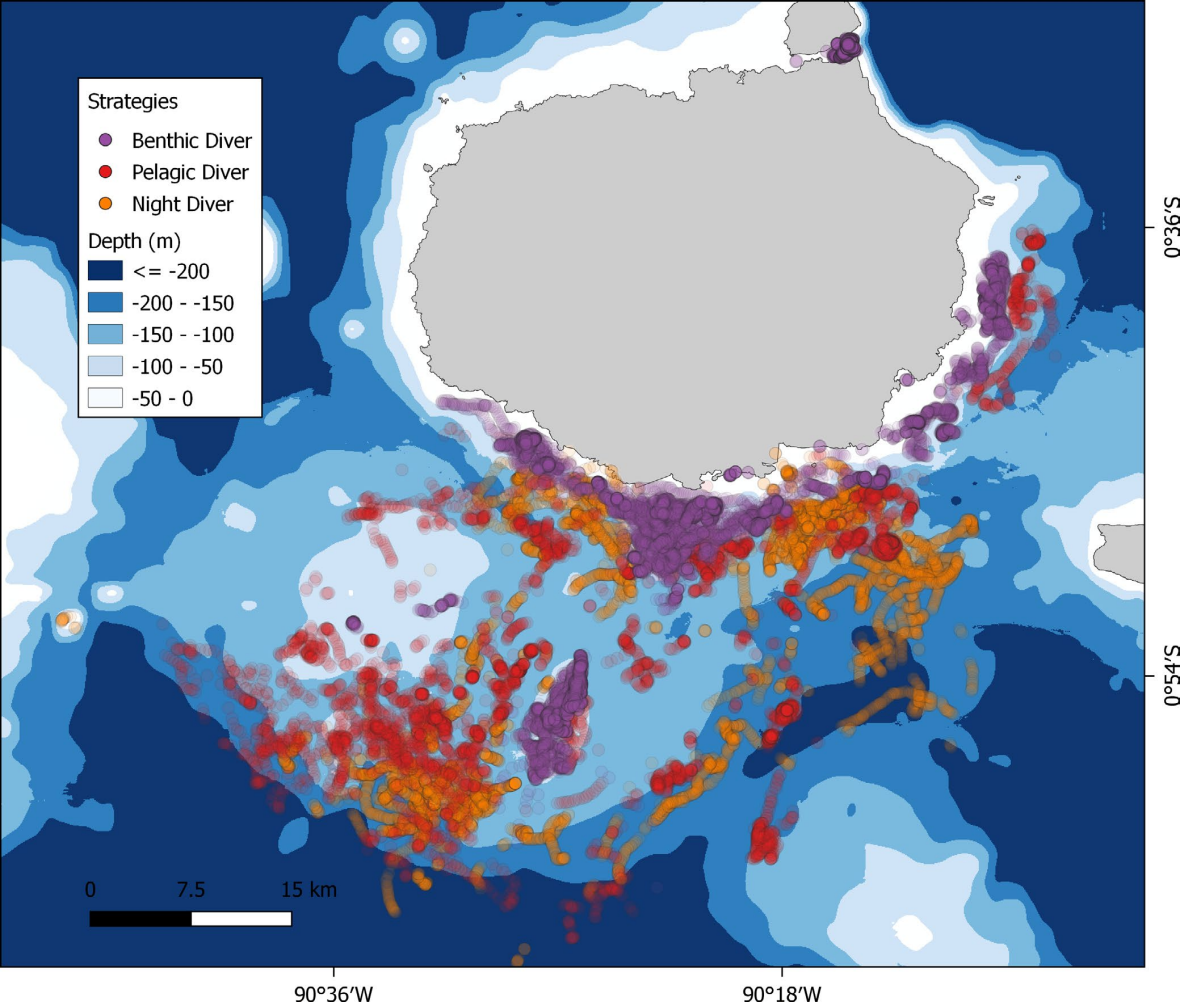
Spatial distribution of foraging strategy clusters on a bathymetric map around Santa Cruz Island, Galapagos. Points are transparent to better visualize GPS locations for individual dives

Trips were clustered based on those variables with a hierarchical cluster analysis using Euclidean distance and Ward's method, revealing three stable clusters. Global Positioning System (GPS) points of dives were also used to identify foraging trips. Figure 2 isolates foraging locations grouped by cluster type, identified as the foraging strategies benthic diver, pelagic diver, and night divers, containing the trips of 12, 12, and 10 individuals, respectively.

### 2.4 Statistical analyses

We first performed hormone data quality assessments to dispel any sampling biases, that is, capture effects. For this, we used R package *lme4* (Bates et al., 2014) in R version 4.0.3 to create linear mixed models (LMMs) and assess relationships between time to sample and hormone values. LMMs included the number of minutes between disturbance and blood collection as a fixed effect, individual ID as a random effect, and each hormonal value was considered as response variables. If values did not appear to significantly change with time, we assumed hormone measurements to reflect near‐baseline values (Romero & Reed, 2005).

We then calculated between‐individual repeatability estimates, or *R*, of hormones using the *rptR* package (Stoffel et al., 2017). An adjusted metric of within‐individual repeatability, *R_i_*, was also calculated using *lme4* package to create LMMs and manually extract individual variance. *R_i_* is the between‐individual variance divided by the sum of the between‐individual plus residual variance for each individual animal (Nakagawa & Schielzeth, 2010). Both estimates are scaled from 0 to 1, from low (0.0–0.4) to high (>0.7) repeatability (Harper, 1994). In addition to repeatability, we also used paired Student's *t* tests to assess the significance and directionality of changes in hormone values that may occur between the initial and return trip measurements. We initially included the number of dives per day, which varied substantially between individuals with a mean of 68 ± 35 dives per day (Schwarz et al., 2021) as a fixed effect to control for foraging effort between initial and return trip measurements, yet this effect was dropped due to lack of significance for each hormone (CORT, *p* = .99; TT3, *p* = .53; TEST, *p* = .18).

Finally, we created separate generalized linear models (GLMs) using the *glm* function to explore how hormone values were associated with foraging group clusters. Pretrip hormone values were entered as predictor variables into separate multinomial logistic regression models with a logit linking function to predict foraging cluster as a categorical response variable. We also controlled for ecologically relevant fixed effects, including age, mass, and pup mass as continuous variables and annual effects (year) as a categorical variable. We based model selection on relative comparisons of fixed effects and their interaction terms according to the lowest corrected Akaike information criterion (AIC_c_). Using stepwise regression with Gaussian distribution, we kept the maximum number of variables while still retaining the lowest AIC_c_, using ΔAIC_c_ >2 as a cut‐off. All data and model residuals were assessed visually for normality and homoscedasticity to meet model assumptions. Alpha values were set at *p* < .05 for significance and trends at *p* < .10. If significant hormonal effects were present, we visualized the magnitude of impact on each foraging cluster using logistic plots of probability curves based on the GLM output.

## 3 RESULTS

### 3.1 Repeatability of hormonal values

**FIGURE 3:**
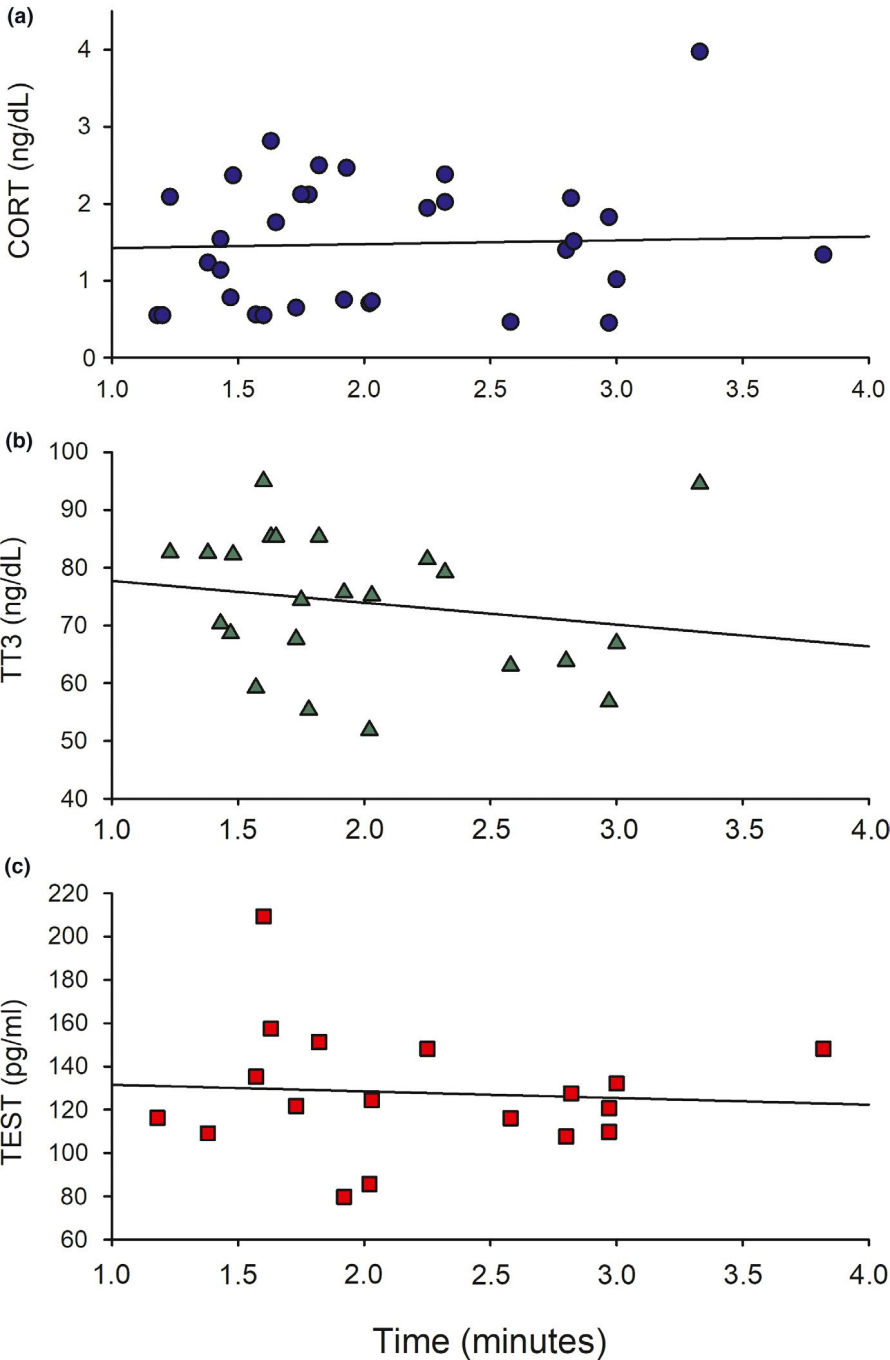
Linear regressions depicting relationship between time postdisturbance and hormone levels in adult female Galapagos sea lions. Time showed no significant linear effect on cortisol (a, CORT), total thyroid T3 (b, TT3), or testosterone (c, TEST)

First, when considering whether hormone levels may have been affected by capture and handling artifacts, we found that time after disturbance had no significant effect on any hormone type (CORT, *R* ^2^ = .01, *p* = .80; TT3, *R* ^2^ = .06, *p* = .26; TEST, *R* ^2^ = .01, *p* = .77). Linear relationships for each hormone are depicted in Figure 3.

Mean hormonal values across all individuals were as follows: CORT = 1.98 μg/dl ± 0.85 *SD*, TT3 = 79.2 ng/dl ± 14.1 *SD*, TEST = 145.2 pg/ml ± 44.0 *SD*. Table 2 shows between‐individual repeatability estimates, or *R*, and within‐individual level repeatability, or *R_i_*, for each hormone type across initial and return trip samples.

**TABLE 2:** Between‐individual repeatability estimates (*R*) for hormone values of cortisol (CORT), total thyroid T3 (TT3), and testosterone (TEST)

	Model	*R*	SE	CI	*R_i_* mean	*R_i_* *SD*
CORT (ng/dl)	Gaussian	0.627**	0.113	0.451–0.878	0.727	0.189
TT3 (ng/dl)	Gaussian	0.589*	0.154	0.219–0.804	0.580	0.231
TEST (pg/ml)	Gaussian	0.899**	0.053	0.760–0.961	0.818	0.122

Note

*R_i_* means and standard deviations (*SD*) represent adjusted within‐individual estimates derived by extracting individual model variances.

Abbreviations: CI, lower and upper 95% confidence intervals; *SE*, standard error.

** Significance of *p* < .001,

* *p* < .05.

**FIGURE 4:**
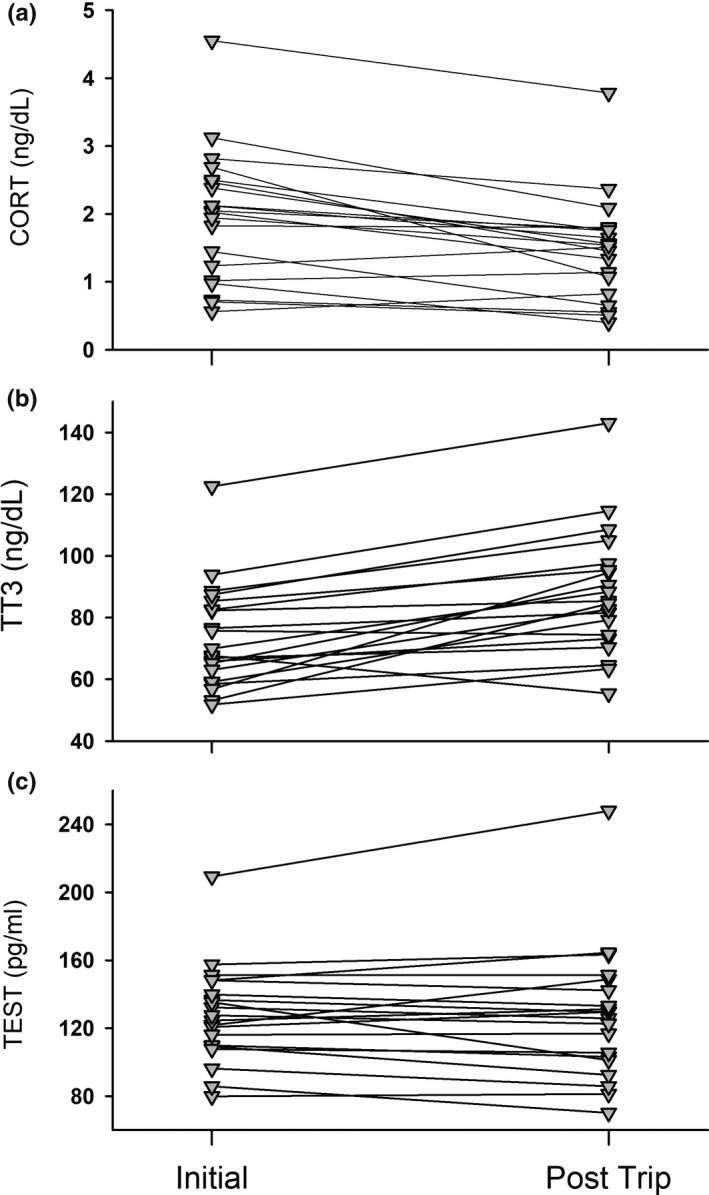
Repeated measurements of near‐baseline cortisol (CORT, a), total thyroid T3 (TT3, b), and testosterone (TEST, c) in adult female Galapagos sea lions (*N* = 20). Solid lines connect initial and post‐trip measurements, separated by a mean of 16 days ± 0.7 *SD* while individuals foraged at sea

To summarize, TEST was strongly repeatable between and within individuals and did not significantly change between both measurement time points (Figure 4, *p* = .95). CORT and total thyroid T3 were only moderately repeatable between and within individuals. Intriguingly, across all animals, initial CORT was significantly higher than return trip values (Figure 4; *R* ^2^ = .17, *F*_1,37_ = 4.10, *p* = .059), while the inverse was observed with TT3 (Figure 4; *R* ^2^ = .19, *F*_1,37_ = 5.89, *p* =.023).

### 3.2 Hormonal variation in relation to foraging type

Within each multinomial logistic model, we found that measured values for each respective hormone type significantly influenced foraging group cluster (Table 3), but the directionality of these effects differed.

**TABLE 3:** Likelihood ratio chi‐squared and *p* values derived from separate generalized linear models describing the influence cortisol (CORT), total thyroid T3 (TT3), and testosterone (TEST) on foraging group cluster type. We controlled for age, mass, pup mass, and annual effects (2018 vs. 2019) in each model

	Cluster type			Cluster type			Cluster type	
	*χ* ^2^	*p*		*χ* ^2^	*p*		*χ* ^2^	*p*
CORT	10.5	.005*	TT3	12.9	<.001*	TEST	13.7	<.001*
Age	0.04	.98	Age	2.66	.26	Age	0.09	.95
Mass	1.61	.45	Mass	1.84	.40	Mass	1.10	.58
Age × Mass	0.15	.93	Age × Mass	1.10	.58	Age × Mass	1.02	.29
Pup mass	0.50	.78	Pup mass	4.13	.13	Pup mass	0.68	.38
Year	4.53	.10	Year	14.9	<.001*	Year	13.7	<.001*

* Significant fixed effects (*p* < .05).

**FIGURE 5:**
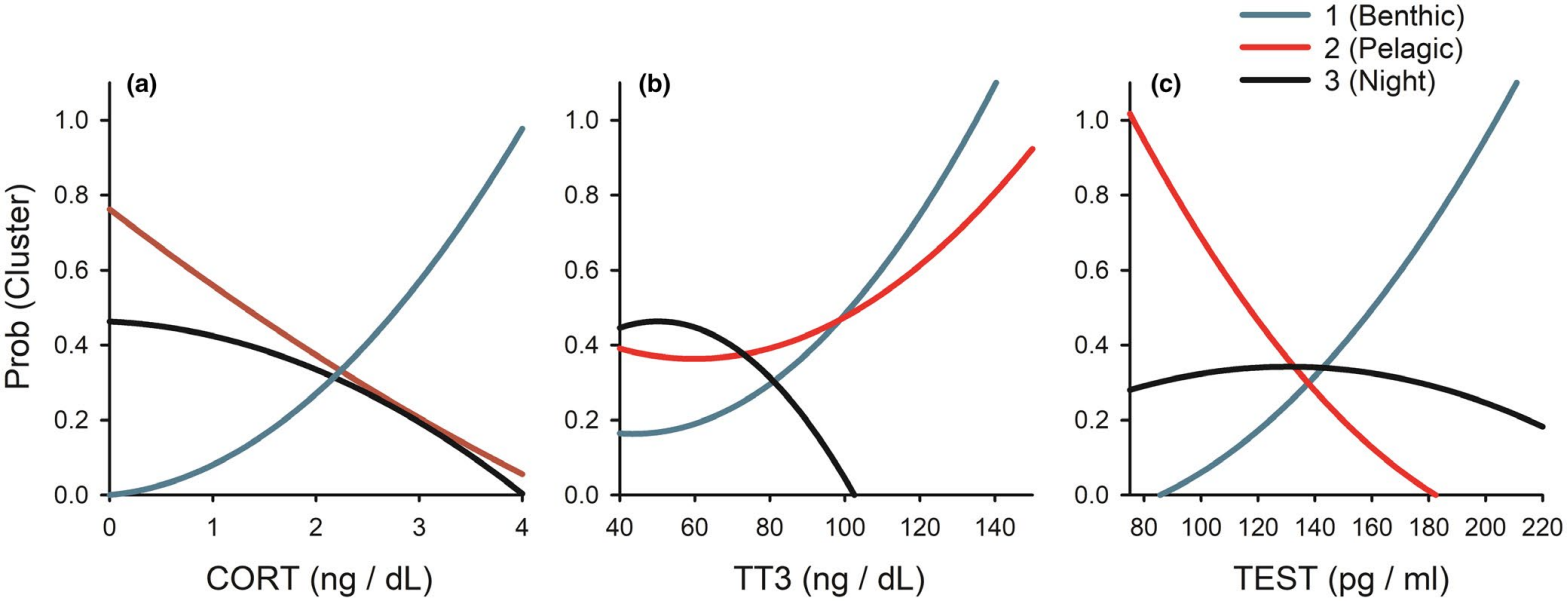
Hormone values as predictors of foraging cluster (*N* = 37). Initial measurements of cortisol (CORT, a), total thyroid T3 (TT3, b), and testosterone (TEST, c) are presented as a function of the probability (Prob) that individuals fall into a foraging cluster, derived from a multinomial regression model

Probability curves visualized from the GLM output revealed strong positive associations between increasing CORT and TEST and the probability of being a benthic diver, while the inverse was observed for pelagic and night divers (Figure 5a,c). Further, night divers stood out from both benthic and pelagic divers, wherein total thyroid T3 (TT3) was significantly reduced in this group than others (Figure 5b). Because we previously found that CORT and TT3 changed within individuals during the study period, a follow‐up analysis using return trip values was also explored in separate GLMs. Results for each hormone type followed similar patterns (likely because individuals changed in predictable ways, that is, CORT increased and TT3 decreased within the study population).

Finally, when considering state effects, year trended toward significance in CORT and TEST models, but was highly significant in the TT3 model (Table 3). Post hoc analysis revealed that a significantly greater number of pelagic divers were observed in 2018 (nine out of 16 animals), while benthic and night divers (eight and 10 out of 22 animals, respectively) were more common in 2019 (*χ* ^2^ = 6.23, *p* = .044). Although there was large variation in female mass (mean ± *SD* = 71.4 ± 8.6 kg, range = 54.3–88.8 kg) and pup mass (mean ± *SD* = 21.4 ± 10.6 kg, range = 6.6–43.4 kg), there was no effect of these variables on diving strategy cluster (Table 3).

## 4 DISCUSSION

In this study, we remotely monitored the at‐sea behavior of GSLs with a specific focus to understand hormonal variation between three distinct foraging strategies. Using this individual‐based approach, we demonstrated moderately high repeatability of near‐baseline CORT, thyroid T3, and TEST values. In line with our prediction, each hormone type had significant yet multidirectional effects on behavioral strategies undertaken by female sea lions during at‐sea foraging. Intriguingly, benthic diving females stood out with predictably higher CORT and TEST levels, while night divers (which generally behave like pelagic divers but at shallower depths and with less duration) were most likely to have low thyroid T3 levels in relation to the other two strategies. Below, we provide context for between‐individual differences and repeatability for each hormone type and raise explanations for how overarching hormonal patterns might modulate behavior indicative of ecological pressures that affect each foraging strategy.

### 4.1 Repeatability of hormonal profiles

The degree of between‐individual variation and within‐individual consistency of hormone levels can be a direct result of environmental, temporal, or contextual differences across sampling periods (Hau et al., 2016; Taff et al., 2018). Here, TEST measurements showed strong between‐individual variation and within‐individual consistency in sea lions which demonstrate high repeatability, as is expected when TEST is measured within same life‐history stages (Ambardar & Grindstaff, 2017; Fanson & Biro, 2019). Less repeatable, however, were CORT and thyroid T3, which changed in predictable directions. CORT was significantly higher during initial samples and lower during recapture. The first capture had a higher likelihood, but not complete certainty, of capturing females about to leave for a foraging trip, while recaptures often happened as soon as they returned on land from a foraging trip. It thereby can be argued that CORT levels were low during post‐tagging and higher during initial measurements. A similar pattern was found in female Antarctic fur seals (*Arctocephalus gazella*), in which CORT was low when individuals returned to pups after a foraging trip yet increased significantly, nearly 67%, toward the end of a 4‐day attendance period (Guinet et al., 2004). This study postulated that CORT may increase as females fast on land while nursing pups, acting as a signal to return to sea to alleviate nutritional stress. Inversely, in our study, T3 increased, which could be attributed to an elevation of active thyroid function to support energy‐intensive behaviors, that is, at‐sea foraging. As such, T3 is often highest at the end of diving trips, such as the case with weaning elephant seals which increase T3 levels by roughly 40% across an 8‐week weaning period while learning to forage (Somo et al., 2015). Here, we show −22% and 20% magnitude changes between initial and post‐trip measurements for CORT and T3, respectively; however, we caution that the timing of a prior foraging trip was unknown before the initial CORT measurement. Therefore, it is yet unclear whether our measurements truly represent pre‐ and postforaging, which would be essential to compare the magnitude of the observed hormonal changes within this context. Therefore, contextual information regarding hormonal measurements, for example, if females spend time on land or foraged at‐sea, should be further studied to understand the biological significance of sources of individual variation which affect overall repeatability, especially for hormones which respond acutely to metabolic needs.

### 4.2 Hormonal effects on energy mobilization?

We initially assumed that hormonal effects on sea lion foraging decisions would be strongly influenced by inherent physiological or metabolic differences between strategies. Studies of energy expenditure in this and other otariid species show that FMRs are associated with an extended time at‐sea, often characteristic of animals which swim to pelagic areas (Villegas‐Amtmann et al., 2017). McHuron et al. (2018) found a similar pattern in the closely related California sea lion (*Zalophus californianus*) between FMR and dive depth and duration, yet in that study, no clear differences in energy intake or expenditure were found between grouped behavioral strategies. Because most studies of pinnipeds consider FMR and few assess the effects of hormonal profiles within a foraging context (reviewed in Atkinson et al., 2015), how these metabolic patterns translated toward hormonal variation between foraging strategies clustered in this study were especially of interest. Here, pelagic divers, with greater depths, durations, and total dive times, had significantly higher thyroid T3 levels than their night diving counterparts. As seen in Schwarz et al. (2021), night divers exhibit quite diverse and unique behavior in that they mostly taking shallow dives of ~30–60 m with shorter durations compared to pelagic divers. Although night divers are known to show flexibility and occasionally take deeper foraging bouts during the day, they mostly exploit mesopelagic prey that overlap with the shallow pelagic zone at night. Therefore, it could be argued that this strategy is energetically conservative or perhaps “low output” compared to pelagic divers by having characteristically lower thyroid levels. This is especially relevant considering that, in true seals, experimentally raised thyroid hormone acutely results in higher oxygen consumption during diving, demonstrating that thyroid may play an activate role in upregulating metabolism during at‐sea foraging (Weingartner et al., 2012). Although it is unclear whether low thyroid represents a causal relationship toward diving or if the behavior itself is regulating thyroid patterns, our result indicates a clear separation between night divers and other groups.

The inverse patterns for levels of CORT and TEST between benthic and pelagic divers (i.e., high in benthic, low in pelagic) were equally intriguing. Because thyroid hormone levels and foraging trip durations are similar between each strategy, it is unclear if metabolic parameters or other factors related to diving difficulty also underlie differences between these two groups. Considering that CORT is responsible for acute energy mobilization, it would be reasonable to predict that pelagic divers, with fast descent rates to reach greater depths, would require higher CORT levels. In addition to strictly energetic requirements, CORT is also associated with oxidative damage that may come from ischemia and hypoxia during breath‐holds across long dives (Crocker et al., 2016). Further, TEST is known to affect locomotory abilities and regulate blood oxygen levels in animals, by promoting myoglobin and overall red blood cell production (Bachman et al., 2014; Mänttäri et al., 2008; Mirand et al., 1965), which as previously mentioned allows for longer foraging durations in this species (Villegas‐Amtmann & Costa, 2010; Villegas‐Amtmann et al., 2012). Therefore, from a strictly mechanistic standpoint, one could presume CORT and TEST would be similar in benthic and pelagic divers as foraging duration was equally similar. However, this was not the case, and interpretation of these patterns may require outside explanations.

### 4.3 Cortisol, TEST, and potential links to risk assessment

In foraging strategies as distinct as those observed in GSL, we know that each group calls for a vastly different behavioral repertoire that allows animals to be successful in their respective niche. Hunting solitary‐living benthic prey by repeatedly utilizing the small relatively shallow prey communities is highly segregated from hunting mobile schooling pelagic fish which must be searched for during each trip anew, and thus may warrant different selective pressures acting on hormone levels. According to risk‐sensitive foraging models, risk‐taking decisions are often related to the unpredictability of resource availability (Toscano et al., 2016), with hormone levels often upregulated when the degree of risk‐sensitivity or effort put toward resource acquisition and defense is stronger. Elevating glucocorticoids like corticosterone, for example, caused mountain chickadees (*Poecile gambeli*) to return more often to known reliable food caches, potentially representing a hormone‐mediated mechanism to buffer the effects of unpredictable environments (Pravosudov, 2003). This closely resembles the benthic diver strategy in the GSL, where benthic divers have higher CORT levels and return repeatably to the same shallow foraging areas which are considered a more reliable habitat but one with lower prey density and quality (Schwarz et al., 2021). Reliability is inferred because the quantity of benthic prey in GSL diets is more common when environmental conditions (e.g., sea surface temperatures) become suboptimal (Páez‐Rosas et al., 2020). Regarding TEST, few examples of its effect on risk‐sensitive foraging exist; however, in one study, elevated TEST levels were demonstrated in white‐eared hummingbirds (*Hylocharis leucotis*) to result in a more risk‐prone strategy, in this case with animals more often visiting flowers with variable rewards rather than reliable ones (Chávez‐Zichinelli et al., 2014). If benthic foraging is indeed reliable, this appears to be opposite to the strategy used by high TEST benthic diving sea lions; therefore, other additional influencing factors on TEST could be at play.

Risk‐taking decisions also involve intra‐ and interspecies interactions during at‐sea foraging that may impact hormonal expression. Due to the particularly confined and sessile nature of benthic divers' prey, it is not unreasonable that high TEST could be a response to increased competition by conspecifics for limited shallow benthic foraging areas. Dense GPS positions of benthic foraging dives around the coastal seabed and on top of the underwater mountain south west from the colony visualized in Figure 1 suggest strong spatial overlap of foraging habitats of benthic divers, especially in relation to the dispersed prey patches used by pelagic and night divers. Being constrained to these areas could promote high TEST levels, considering that TEST facilitates territorial defense among conspecifics (Mehta & Josephs, 2010; Wingfield et al., 1990). Similar correlations of foraging habitat density and TEST have been observed in grizzly bears (*Ursus arctos*), in which bears living in coastal habitats with high conspecific overlap for spatially constrained salmon resources also had the highest TEST levels when compared to more dispersed, generalist bears (Bryan et al., 2013). It should also be noted that, specific to the Galapagos archipelago, the highest abundance of shark species which prey on sea lions are found around near‐shore islets and seamounts (Acuña‐Marrero et al., 2018; Salinas‐de‐León et al., 2019), strongly overlapping with the foraging areas of benthic divers. TEST is a known inhibitor of apprehension toward predators in a variety of contexts (King et al., 2005; Wingfield et al., 2001). Long‐term risks and costs associated with social competition and predator interactions in each marine habitat type are currently speculative; however, future efforts could elucidate these factors to understand the trade‐offs and selective pressures that may be driving hormonal, and ultimately, fitness differences associated with foraging types.

Finally, we show that an annual effect on foraging cluster approached significance or was present in T3 and TEST models, wherein pelagic diving was a more common strategy for individuals in 2018 compared to 2019. This effect requires caution, as it could be due to a random bias in our relatively narrow sample size across only two years or may reflect annual differences in marine productivity affecting foraging decisions (Páez‐Rosas et al., 2020; Urquía & Páez‐Rosas, 2019). It is worth noting that 2018 was a year with higher annual mean surface temperatures in comparison to 2019, as measured by the Charles Darwin Research Station near Caamaño islet (Charles Darwin Foundation, 2021). High temperatures are known to drive pelagic prey species offshore, thus making this strategy more difficult and inducing nutritional stress (Trillmich et al., 2014). From a hormonal standpoint, it has been shown that CORT and thyroid are reduced during nonideal conditions, as observed in California sea lions (*Z. californianus*) during a recent El Niño event in 2015 (DeRango, Prager, et al., 2019). Thus, these results leave the stage open for examining broader individual patterns of hormonal and behavioral adjustments, especially those which span across a greater number of years or degrees of fluctuating environmental conditions.

## 5 CONCLUSION

In summary, we offer a novel perspective on understanding between‐individual variation in foraging behavior and its hormonal correlates in a large marine predator. Female sea lions showed moderately strong between‐individual differences and within‐individual repeatability in hormone levels. Additionally, CORT and TEST levels were higher in females using benthic diving strategies, while night divers differentiated themselves from pelagic divers by having characteristically low thyroid T3 levels. We propose that factors such as energetic demands may be driving observed differences in hormone levels between foraging strategies, but also that hormonal phenotypes might facilitate different behavioral repertoires in animals based on qualities of the environment and/or competition among conspecifics. In this context, the extent of the role that hormones might play in risk assessment and social dynamics while foraging are two understudied features in pinniped diving physiology research. Therefore, our results highlight the collective effects of hormonal and ecological variation on specialization in foraging strategies, which should help guide future research that seeks to understand the proximal mechanisms that may underlie variation in adaptive behavior and individual niches in wild populations.

## CONFLICT OF INTEREST

We declare we have no conflicts of interest.

## AUTHOR CONTRIBUTION

**Eugene DeRango:** Conceptualization (equal); Data curation (equal); Formal analysis (equal); Methodology (equal); Project administration (equal); Writing‐original draft (lead); Writing‐review & editing (lead). **Jonas Schwarz:** Conceptualization (equal); Data curation (equal); Formal analysis (equal); Funding acquisition (supporting); Investigation (equal); Methodology (equal); Writing‐original draft (equal); Writing‐review & editing (equal). **Paolo Piedrahita:** Writing‐review & editing (supporting). **Diego Paéz‐Rosas:** Investigation (supporting); Project administration (supporting); Writing‐review & editing (supporting). **Daniel E Crocker:** Formal analysis (supporting); Methodology (supporting); Resources (supporting); Supervision (supporting); Writing‐review & editing (supporting). **Oliver Krüger:** Conceptualization (equal); Data curation (equal); Funding acquisition (lead); Investigation (equal); Project administration (lead); Supervision (lead); Writing‐original draft (supporting); Writing‐review & editing (equal).

## Data Availability

Data available from the Mendeley Data repository: http://dx.doi.org/10.17632/5gh8n56xcs.2, DeRango (2021).
